# Recurrent Submandibular Sialolithiasis in a Child

**DOI:** 10.7759/cureus.12163

**Published:** 2020-12-19

**Authors:** How Kit Thong, Hafiz Mohamad Mahbob, Primuharsa Putra Sabir Husin Athar, Tengku Mohamed Izam Tengku Kamalden

**Affiliations:** 1 Otolaryngology - Head and Neck Surgery, Hospital Sultan Ismail, Johor Bahru, MYS; 2 Otolaryngology - Head and Neck Surgery, KPJ Healthcare University College, Nilai, MYS

**Keywords:** salivary gland calculi, submandibular gland, sialolithiasis, pediatric, sialadenitis

## Abstract

Sialolithiasis is a commonly encountered disease of the salivary glands, reported to represent up to 30% of all salivary gland diseases. However, the condition is rarely encountered in the pediatric population. The formation of a salivary stone is believed to be secondary to the deposition of calcium salts around a nidus. The formation of a nidus is commonly associated with desquamated epithelial or sloughing from a recent bacterial infection. Patients with submandibular sialolithiasis usually present with acute swelling over the neck associated with pain, fever, and purulent intraoral discharge. Neglected and poorly treated acute infection may progress to life-threatening abscess formation. Here we are describing our encounter with a 10-year-old boy with recurrent submandibular sialolithiasis. He was initially treated with conservative measures and antibiotics regimen. Failure of medical treatment and recurring symptoms led to submandibular gland excision followed by a full recovery.

## Introduction

Sialolithiasis is commonly seen in the adult population, representing up to 30% of all salivary gland diseases [1]. However, the prevalence of sialolithiasis in children is much rarer and only accounts for 3% of the cases [2]. Sialolithiasis is a condition that involves the formation of calcareous deposits within a salivary gland or its excretory duct causing obstruction to normal salivary flow. Consequently, the mechanical obstruction causes stasis of salivary flow and formation of sialectasis, which predisposes retrograde infection from the oral cavity in the gland [2]. The formation of a salivary calculus is believed to be secondary to the deposition of calcium salts around a nidus which can be caused by desquamated epithelial or sloughing from a recent bacterial infection [3]. Sialolithiasis is an important diagnosis to be excluded in children with recurrent salivary gland infections as it is a treatable condition. Obstructions of the salivary ducts may lead to inflammation from destructive salivary enzymes and superimposed bacterial infection, or in rare cases abscess formation. Early diagnosis and prompt treatment are vital to reduce the morbidity of such patients, especially in the pediatric age group. We present a rare case of pediatric recurrent sialolithiasis that required submandibular gland excision in a 10-year-old boy. We will discuss the clinical presentation, radiographic findings, and particularly the surgical management after the failure of medical treatment.

## Case presentation

A 10-year-old male patient presented to our outpatient clinic with complaints of fever and a painful submandibular swelling for the past two days, the pain is precipitated with food intake. Further history revealed that this is the third episode in the past two years. Previous episodes were resolved with a prescription of oral antibiotics and analgesics from a local clinic. He had no other underlying health issues. Physical examination revealed a submandibular swelling measuring 5 x 4 cm, the submandibular area appeared inflamed and was tender to touch. Upon intraoral examination, a solid mass was felt over the right floor of the mouth area, possibly representing the calculus, and purulent pus discharge was also noted at the right submandibular duct opening. Other systemic examinations were unremarkable. In addition, he had a fever with a recorded temperature of 38.5’C. Laboratory investigations revealed leukocytosis (white blood cells, 14.2x10^9^/L) and C-reactive protein level at 30.2 mg/dL. Serum uric acid level, total serum calcium, and corrected calcium level were normal. The patient was started on empiric intravenous antibiotics and oral analgesics consisting of paracetamol and ibuprofen. Ultrasound of the neck was performed the next day revealing dilatation of the right submandibular duct, measuring 0.2 cm in diameter, with calculi measuring 0.4 x 1.2 cm lodged in the distal Wharton’s duct. Abdominal ultrasound was negative for calculus in the kidneys, gallbladder, and pancreas. Swelling and pain resolved after day 3 of intravenous antibiotics. He was discharged from the ward with oral antibiotics for a week.

**Figure 1:**
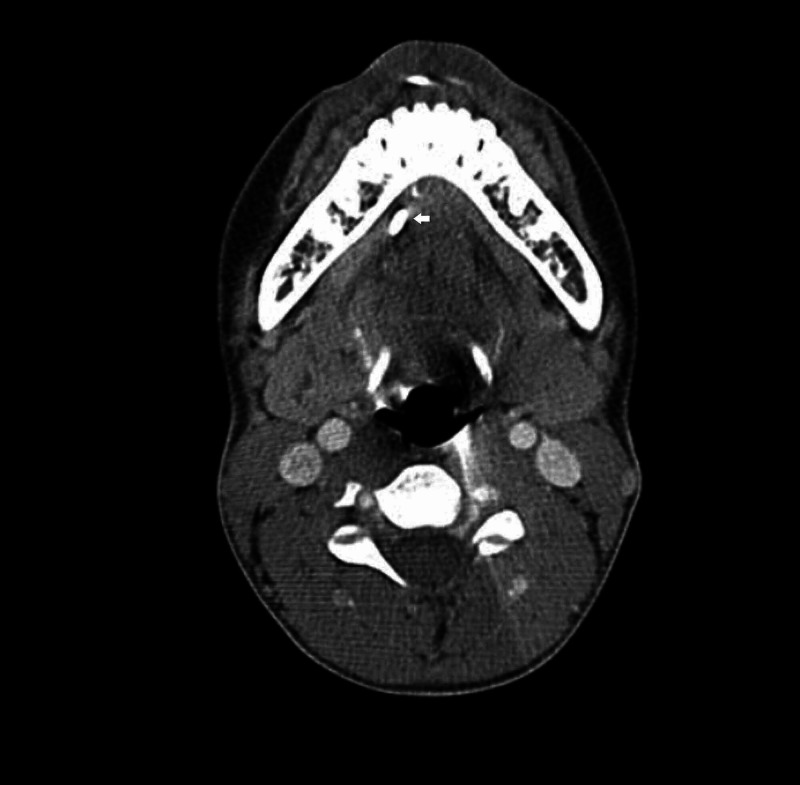
CT scan of the neck (axial view) with a stone (white arrow) seen in the Wharton's duct

**Figure 2:**
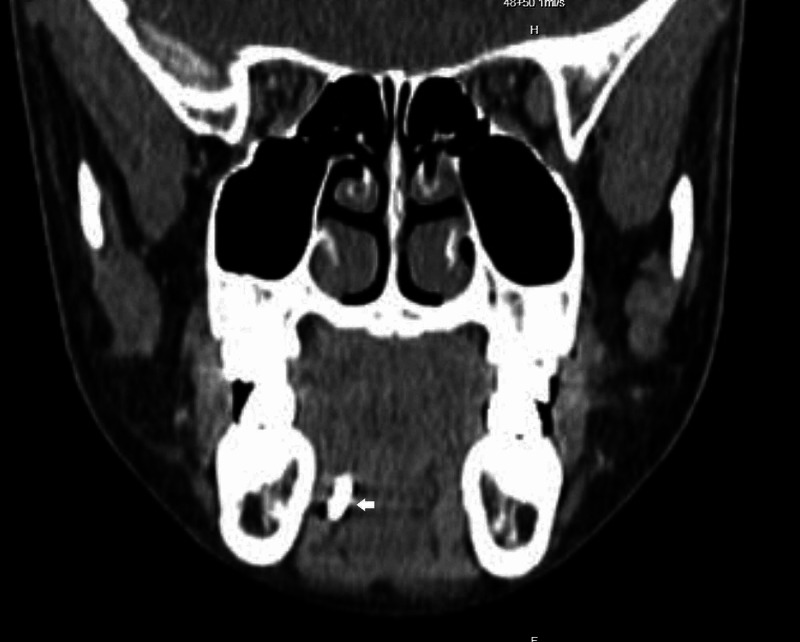
CT scan of the neck (coronal view) with a stone (white arrow) seen in the Wharton's duct

On subsequent outpatient follow-up two weeks later, the patient reported that the passing of the sialolith had occurred one week after being discharged from inpatient care. The patient was seen during follow-up at our ENT outpatient clinic for two more visits over a six-month period in which he reportedly had another episode of submandibular swelling, with passing of sialolith after being treated with oral antibiotics at a local community clinic. Due to the recurrent nature of symptoms and sialolith formation, a computed tomography (CT) scan of the neck was scheduled. The CT scan revealed multiple sialoliths, a 0.8 x 0.4 cm calculus situated within the proximal right submandibular duct, and a smaller calculus was also seen distally, measuring 0.3 x 0.2 cm. The right submandibular duct was dilated (Figures 1-2). The patient was then scheduled for a submandibular gland excision with an indication of recurrent right submandibular duct sialolithiasis. The surgery was uneventful and there was no complication in the postoperative period. Intraoperatively, the gland removed was smooth and normal in appearance, with a size measuring 4 x 3 cm. Histopathological examination of the specimen reported Iymphoplasma cell aggregates accompanied by fibrosis features suggesting chronic sialadenitis. The patient was seen during follow up at our outpatient clinic for six months after surgery and there was no recurrence of neck swelling or sialolith formation.

## Discussion

Sialolithiasis is a relatively common salivary gland disease in adults with an estimated 12 in 1,000 of the population. However, it is an extremely rare occurrence in the pediatric population, accounting for only 3% of all sialolithiasis [1,2]. The disease has a few associated pathogenetic factors which include salivary flow obstruction, decreased salivary flow, and altered salivary pH secondary to dental and oropharyngeal infection. Multiple theories have been proposed in relation to the pathogenesis of salivary stone formation, but most remain controversial and inconclusive. Marchal et al. proposed a retrograde theory for sialolithiasis formation which involves retrograde migration of food particles, foreign bodies or pathogens ascending from the oral cavity into the salivary ductal system, which subsequently act as a nidus for further calcification resulting in salivary stone formation [4]. The retrograde theory by Marchal et al. was felt to be the most relevant by the authors. Submandibular stones are more commonly encountered due to their anatomical predisposition of longer and narrower drainage duct, slower salivary drainage due to its flow against gravity, saliva pH being more alkaline and containing a higher concentration of phosphate, calcium, and mucinous proteins contents [5]. With the exception of gout, there is no proven association of other systemic diseases and sialolithiasis [6]. Patients with submandibular sialolithiasis usually present acutely with painful and inflamed swelling accompanied by intraoral purulent discharge or it may present as a neck abscess. The symptoms are often aggravated by oral intake. Various imaging modalities may aid the diagnosis of sialolithiasis. Conventional pain radiography may be used to diagnose sialolithiasis but its application is limited, as it may be able to visualize only 60% of the parotid and 80% of submandibular calculi [2]. Sialography with contrast is a useful alternative to outline ductal anatomy. But as in our case, it is relatively difficult to perform in uncooperative children and it may further worsen the acute infection [7]. The stone is described as a filling defect within the salivary duct or gland [7]. Other imaging modalities including CT scan and magnetic resonance imaging (MRI) should also be considered as both have high accuracy and are non-invasive, with CT scan being more cost effective. Similarly in our patient, CT scan was able to accurately localize multiple calculus and delineate the salivary gland anatomy. Sialoendoscopy is an upcoming procedure that can be utilized either as a diagnostic or therapeutic option [8].

The first line of treatment should be conservative by ensuring good hydration, frequent hot compresses, sialogogues, analgesics, and antibiotics. Previous reports indicate that smaller stones with a dimension less than 2 mm may pass out spontaneously in adults [9]. However, spontaneous passing may be difficult in children due to the smaller ductal opening [3]. More invasive modalities include submandibular gland excision. Submandibular gland excision is mainly reserved for recurrent cases or intraparenchymal sialolithiasis which can be achieved either by means of transcervical or intraoral approach [10]. Intraoral submandibular gland excision has been described previously, such an approach has an advantage of external scar avoidance and a lowered risk of hypoglossal and marginal mandibular nerve injury [11]. In our case, the submandibular gland excision was performed due to the proximally located ductal stones and recurrent formation of sialolithiasis. Removal was done through the transcervical route as the authors found it difficult and challenging to perform transoral excision in pediatric patients due to the limited surgical field and higher complication rate of saliva fistulas, salivary gland remnant, postoperative submandibular swelling, and hemorrhage [12].

## Conclusions

In conclusion, although sialolithiasis is a rare occurrence in the pediatric population, it should not be missed in the differential diagnosis of pediatric patients with submandibular swelling and pain. A high index of suspicion should be maintained, as a delayed diagnosis may cause significant discomfort and morbidity to the patient. Medical therapy is the mainstay of treatment, however, there is a role for submandibular gland excision in selected cases as presented here.
